# The Predictive Value of Return to Work Self-efficacy for Return to Work Among Employees with Cancer Undergoing Chemotherapy

**DOI:** 10.1007/s10926-020-09882-2

**Published:** 2020-02-29

**Authors:** Rikke Rosbjerg, Dorte Gilså Hansen, Robert Zachariae, Inger Hoejris, Thomas Lund, Merete Labriola

**Affiliations:** 1 Department of Public Health, Aarhus University, Aarhus, Denmark; 2 DEFACTUM, Central Denmark Region, P.P. Ørums Gade 11, 1.B, 8000 Aarhus C, Denmark; 3 Research Unit of General Practice, Institute of Public Health, University of Southern Denmark, Odense, Denmark; 4 Unit for Psychooncology and Health Psychology, Department of Oncology, Department of Psychology, Aarhus University Hospital, Aarhus University, Aarhus, Denmark; 5 Department of Oncology, Aarhus University Hospital, Aarhus, Denmark; 6 Centre for Social Medicin, Frederiksberg and Bispebjerg Hospital, Copenhagen, Denmark; 7 Department of Public Health, University of Copenhagen, Copenhagen, Denmark; 8 NORCE Norwegian Research Centre AS, Bergen, Norway

**Keywords:** Return to work, Work ability, Self-efficacy, Cancer, Prediction

## Abstract

*Purpose* The aim of the present study was to examine the predictive value of Return to Work Self-efficacy (RTWSE) on Return to Work (RTW) among employees undergoing chemotherapy for cancer and to examine the relative contribution of RTWSE as predictor variable compared to personal, health-related, illness- and treatment-related and work-related factors. *Methods* A sample of 114 sickness absent employees with various cancers (age 18–62) included in the study on average 33 days after initiating chemotherapy were followed for 15 months. Data sources included patient questionnaires (RTWSE, depression, fatigue, performance status), sociodemographic factors (age, sex, job type, and perceived support from the workplace), patient records (type of cancer, treatment intention, number of treatment modalities, time since diagnosis and time since initiation of chemotherapy), and Danish national registries (RTW and education). Associations between RTWSE at baseline and weeks until full RTW during 15-months follow-up were analyzed using Cox proportional hazards regression. *Results* In the univariate analysis, high RTWSE was associated with shorter time to RTW (Hazard Ratio (HR) 1.84, 95% confidence interval (CI) 1.12–3.03). In the multivariate model, RTWSE failed to reach statistical significance (HR 1.12, 95% CI 0.62–2.02), whereas female sex (HR 0.30, 95% CI 0.15–0.60) and receiving palliative treatment (HR 0.15, 95% CI 0.05–0.44) were significantly associated with later RTW. *Conclusion* Compared to other factors of significance, RTWSE was not the strongest predictor of RTW when examined among employees undergoing chemotherapy for cancer. Before using the RTWSE questionnaire to identify employees with cancer at risk of late RTW, it is important to recognize that the predictive value of RTWSE may be different for employees on sick leave due to cancer than for other sickness absence populations.

## Background

Employees with previous or current cancers have an increased risk of sickness absence [1–3], unemployment [4], reduced work ability [1, 2, 5], and early retirement [6, 7] compared to the general population. In Europe, the annual incidence of cancer is 4.2 million cases [8]. Of these, approximately 50% are in the working age [4, 9]. Due to increasing incidence [8], as well as improved treatments, the number of cancer survivors has been steadily increasing during the last decades, i.e., 12.1 million individuals in Europe in 2018 [8]. Working life is an important aspect of most people's identity, role functioning, mental health, and quality of life [10, 11]. For cancer patients, work furthermore represents a possibility to get emotional support outside the family and to capture aspects of normal life [12–14]. Thus, to most people, job loss and sickness absence have considerable negative consequences [11]. Furthermore, the financial burden of work disability due to cancer is high for society [15, 16]. Hence, improving the work ability and the process of return to work (RTW) for the steadily increasing number of people with current or previous cancers is of major importance.

In occupational rehabilitation research, self-efficacy (SE), defined as an individual's belief in his or her own ability to undertake behaviours to achieve specific desired goals [17], has been identified as an important psychological factor in the RTW process among employees with various health problems [18–21]. Measuring return to work SE (RTWSE), several RTWSE questionnaires have been developed. Based on qualitative interviews with employees on sick leave due to work-related low back pain, Shaw et al. [20, 21] developed and validated a 19-item RTWSE questionnaire (RTWSE-19). In 2010, an 11-item RTWSE questionnaire (RTWSE-11) was developed and validated by Lagerveld et al. [19] in a population of employees with mental health problems and in 2011, a ten-item RTWSE questionnaire (RTWSE-10) was developed and validated by Brouwer et al. [22] in a population of employees with musculoskeletal disorders.

RTWSE has been shown to be positively associated with work ability and employment status [20, 21] and has further proven to be a strong predictor of actual RTW in various sickness absence populations [23, 24]. Shaw et al. [20] showed RTWSE-19 to be predictive of actual RTW at 1-week follow-up and at 3-month follow-up in a population of sickness absentees with low back pain. Using the RTWSE-11, RTWSE has furthermore been found predictive of actual RTW among employees with mental health problems within 3 months after baseline [19] and within 12 months after initiation of sick leave [25]. By use of the RTWSE-10, Brouwer et al. [23] found RTWSE to be predictive of RTW at 6-month follow-up in employees on sick leave due to musculoskeletal disorders. The predictive value of RTWSE has also been shown in samples of all-cause sickness absence, using the RTWSE-19 [26] and the RTWSE-11 [24].

Thus, the predictive value of RTWSE has been confirmed in several populations. Little attention, however, has been given to the predictive value of RTWSE in populations of cancer patients [27, 28]. As cancer is a potential life threatening disease and studies have shown that a cancer diagnosis may change life priorities and the meaning of work [1], the predictive value of RTWSE may be different compared to other sickness absence populations. To our knowledge, the predictive value of RTWSE in a cancer population has been examined only once, using the RTWSE -11, and as part of an intervention study in which RTWSE showed to be predictive of full RTW during 18 months of follow-up [29]. This finding was based on a sample of 81 patients with cancer (mainly breast cancer, 87%), undergoing chemotherapy with a curative intention, participating in an intervention program aiming at increasing RTW among cancer patients [29]. In order to generalize the results from that study, one should notice that the participants were all (I) in curative treatment, (II) physically well-functioning, and (III) had chosen to participate in an intervention program aiming at enhancing RTW. Hence, both the physical functioning and the motivation for RTW may have been higher in this population than in a general population of employees with cancer. The predictive value of RTWSE for employees with cancer is thus scarcely examined.

The primary aim of the present study was therefore to examine the predictive value of RTWSE on full RTW during 15 months of follow-up in a sample of sickness absent employees undergoing chemotherapy for various cancers.

Systematic reviews have shown that RTW of people with current or previous cancers is associated with multiple factors [1, 3, 30–32]. The following factors have repeatedly been shown to be of significance in the RTW-process of cancer patients: personal factors (e.g., socioeconomic factors), health-related factors (e.g., physical and mental health), illness- and treatment-related factors (e.g., type of cancer, type of treatment), and work-related factors (e.g., work demands, type of work, working environment) [1–3, 32]. A secondary aim of the present study was to examine the relative contribution of RTWSE as predictor variable compared to personal, health-related, illness- and treatment-related, and work-related factors.

## Methods

### Study Design and Setting

The predictive value of RTWSE was examined in a prospective longitudinal study in which employees with various cancers, initiating chemotherapy at Aarhus University Hospital, Denmark, between November 2016 and May 2018, were invited to participate in a survey study about physical activity and work status [33]. Data sources included patient questionnaires, patient records, and Danish national registers. In the present study, only employees on full time sick leave at baseline were included and followed for 15 months after baseline.

### Participants

#### Inclusion Criteria

The inclusion criteria were: (I) 18–62 years of age; (II.a) initiating chemotherapy for a newly diagnosed cancer disease; or (II.b) due to relapse, if the patient had not initiated chemotherapy for a previous or current cancer during the last 24 months; (III) all treatment intentions (i.e., curative, palliative, adjuvant and neo-adjuvant); (IV) employed at the time of inclusion, but on full time sick leave at baseline; (V) time of follow-up ≥ 15 months; and (VI) ability to read and understand Danish.

#### Procedure

A stepwise inclusion procedure was followed. First, the Clinical Trial Unit at Aarhus University Hospital identified eligible patients with regard to age and history of cancer. At the initiation of the first chemotherapy cycle, a clinical nurse gave eligible patients a short oral introduction to the study and a package including a study information folder, a contact sheet (including two options of which the patient was asked to mark one; either a consent for receiving a phone call from a research assistant to learn more about the study or a decline to participate in the study), and a written informed consent (final consent for participating in the study). At the first chemotherapy session, some patients were considered incapable of receiving information regarding the project. In these cases, the nurses postponed the information until the second or third session. Time gaps between sessions varied according to the different treatment plans. Patients who were interested in learning more about the project signed the contact sheet allowing a research assistant to contact the patient by telephone. On the phone, the research assistant screened the patients regarding employment status and provided additional information about the project. Written informed consent was retrieved from those who were eligible and wanted to participate. Subsequently, a baseline questionnaire was sent by e-mail or regular mail in accordance with the patient's preference. Finally, all patients who returned the baseline questionnaire were included. In case of no response, two reminder e-mails were sent after 5 and 10 days, respectively [33].

#### Study Sample

**Fig. 1:**
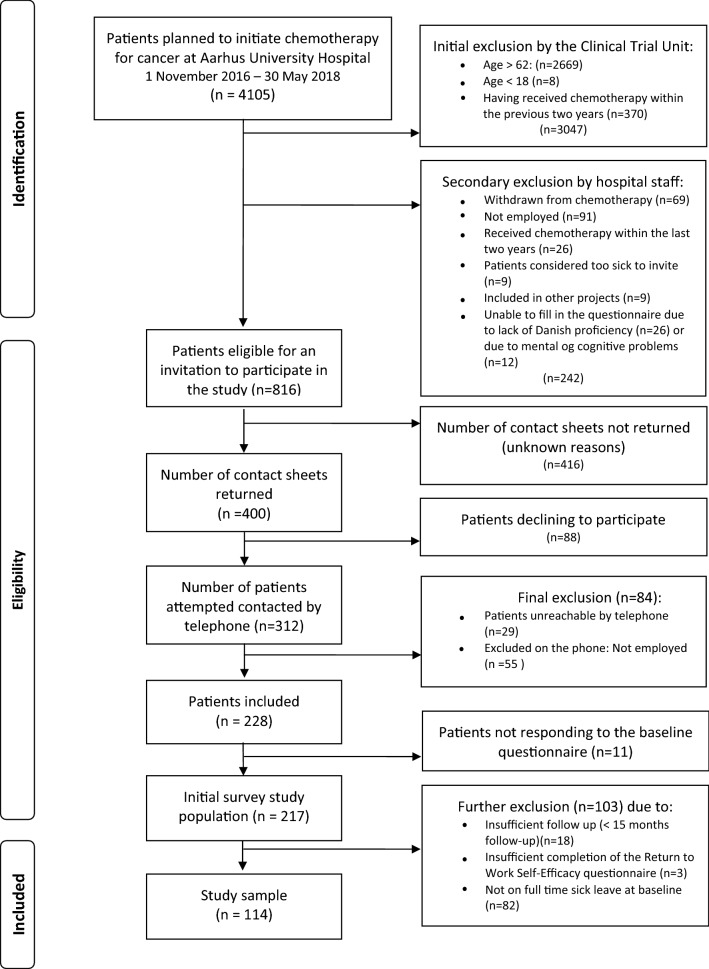
Flow chart of inclusion

From the original survey study population of 217 employees undergoing chemotherapy for cancer [33], 135 were on full time sick leave at baseline including 114 (84%) with 15 months of follow-up and complete data for the present study (see Fig. 1).

### Variables of Interest

#### Dependent Variable

##### Return to Work

Data regarding RTW was obtained from "The Danish Register for Evaluation of Marginalization" (DREAM), a national register containing information on all public transfer payments administered to citizens in Denmark since august 1991 [34, 35]. According to the current Danish law [36], all citizens who are not able to work due to physical or mental disabilities are entitled to receive public transfer payments, e.g., sickness absence compensation or early retirement. Receiving sickness absence compensation is possible after 4 weeks of sickness absence and for 22 weeks in total. An extension of the sickness absence period for an additional 26 weeks is possible for individuals with life-threatening illnesses. Cancer patients are therefore often entitled to this extension. The public transfer payments are registered with a three-digit code on a weekly basis if a citizen receives public transfer payment for one day or more pr. week. If no public transfer payment is registered in a given week, the citizen is regarded as self-supported [34, 35]. The DREAM database has been found to be a valid tool for research of sickness absence and RTW [37]. In the present study, the dependent variable was number of weeks until full RTW and was defined as the first week of at least four successive weeks without public transfer payment (i.e., being self-supported). The four-week window is in line with previous studies of the predictive value of RTWSE [24, 25]. With a 15-months (i.e., 65 weeks) follow-up period and the four successive weeks window, RTW could thus range from 1 to 62 weeks.

### Independent Variables

All independent variables were obtained at baseline.

#### Return to Work Self-efficacy (RTWSE)

The primary independent variable was RTWSE which was measured by *the RTWSE-19 questionnaire*, including 19 statements concerning a person’s belief in his or her own ability to handle different aspects of returning to work [20, 21]. For each statement, the participants rated their confidence on an 11-point numerical rating scale from 0 (”not at all certain”) to 10 (”completely certain”). The questionnaire includes a total scale and three subscales: “Meeting job demands” (7 items), “Modifying tasks” (7 items), and “Communicating needs” (5 items). The questionnaire has shown high internal consistency in a Danish population: 0.93 (“Communicating needs”), 0.94 (“Modifying tasks”), and 0.97 (total scale and “Meeting job demands”) [26]. In the present study, only the total mean score was used. Total mean score was calculated by dividing the total sum by the number of completed items and ranged from 0 to 10 with higher scores indicating better RTWSE. As originally defined by Shaw [20], scores < 5, scores between 5 and 7.5 and scores > 7.5 were considered as low, moderate and high RTWSE, respectively. In the present study, the scale was dichotomized at 7.5. Hence, persons scoring ≤ 7.5 were considered low in RTWSE and persons scoring > 7.5 as high in RTWSE. According to guidelines [38], a total score was considered missing in case of > 20% missing values.

#### Health-Related Variables: Depression, Fatigue and Performance Status

Depression was assessed with the 21-item *Beck’s Depression Inventory* [39]. The total sum score of the scale ranges from 0 to 63 and was further categorized into the following: No depression (0–13), mild depression (14–19), moderate depression (20–28), or severe depression (29–63). The BDI is a widely used valid measure of depression [40] with a Cronbach's alpha value of 0.90 found in a population of women with breast cancer [41]. Missing items were handled by replacement with the average, i.e., mean imputation, but only in cases with no more than 50% missing items and with high internal consistency (Cronbach's alpha > 0.70).

Fatigue was measured with the 13-item *Functional Assessment of Chronic Illness Therapy-Fatigue (FACIT-F)*, version 4, which assesses fatigue and its impact upon daily activities and function during the previous 7 days. Each item is scored on a five-point scale: 0 (not at all), 1 (a little bit), 2 (somewhat), 3 (quite a bit), and 4 (very much). The total sum score ranges from 0 to 52, with higher scores indicating higher fatigue. The scale has demonstrated good validity and reliability in populations of patients with cancer and has shown Cronbach's alpha values > 0.93[42, 43]. Missing items were handled as described in the guidelines of the FACIT-F [44].

Performance status was measured by the 1-item performance status index, developed by *The Eastern Cooperative Oncology Group* (ECOG) [45], in which the participants categorized them selves in one of five levels of performance: (0) Fully active, able to carry on all pre-disease performance without restriction; (I) restricted in strenuous activity but able to carry out work of a light nature; (II) capable of all self-care but unable to carry out any work activity, up and about for more than 50% of the time; (III) capable of only limited self-care, in bed for more than 50% of the time, or (IV) cannot carry out any self-care, totally confined to bed or chair.

#### Illness- and Treatment-Related Variables

The following factors were obtained from patient records by an oncologist: type of cancer, treatment intention (curative, palliative, adjuvant, neo-adjuvant), number of treatment modalities in addition to chemotherapy, time since diagnosis (days), and time since initiation of chemotherapy (days).

#### Work-Related Variables

The participants reported job type (sedentary, physical, mixed) and the degree of perceived support from the workplace on an ad hoc 10-point numerical rating scale (NRS), with 10 indicating the highest level of perceived support.

#### Sociodemographic Variables

Information regarding age, sex, and education was obtained by self-report. Information regarding education was also obtained from the Danish Education Register at Statistics Denmark and categorized into four levels based on the highest level of completed education: (1) None: < 10 years of education (compulsory school), (2) Short: 10–12 years of education, (3) Moderate: 13–15 years of education, and (4) Long: > 15 years of education [46]. In case of missing information regarding education in the register, self-reported information regarding educational level was used.

### Statistics

Baseline characteristics were presented as frequencies and percent or by means and standard deviations. For non-normally distributed variables, median and interquartile range (IQR) were reported. The median number of days until full RTW was calculated for the low RTWSE group and the high RTWSE group, respectively. For both groups, Aalen–Johansen cumulative incidence curves for full RTW during 15 months of follow-up were calculated as well. Permanent exit from the labour market (i.e., retirement) and death were categorized as competing events.

Using a Cox proportional hazards regression, unadjusted and adjusted hazard ratios (HR) were calculated with RTWSE as the independent variable and weeks to full RTW as the dependent variable. A HR value > 1 indicated shorter time to RTW. The proportional hazards assumption was evaluated by use of log-minus-log survival curves and by observed and fitted survival curves.

First, an unadjusted Cox proportional hazards regression was performed, model 1. In the next step, the associations between each of the independent variables and the dependent variable (i.e., RTW) were examined by bivariate Cox proportional hazards regression analyses. Independent variables associated with the dependent variable at a statistically significant level of p < 0.20 were subsequently included in the multivariate models according to the following plan: In model 2, the sociodemographic variables were added, in model 3, the illness- and treatment-related variables were added, and in model 4, the health- and work-related variables were added. Death and permanent exit from the labor market were considered competing events. The significance level of p < 0.20 to identify potential other predictor variables was chosen to prevent exclusion of important variables, and thereby overlooking potential predictor variables in the following models. This procedure is in line with the procedure in a previous similar study [24].

The independent variables finally included in the multivariate model were checked for multicollinearity by the variance inflation factor (VIF). A VIF value > 5 indicates multicollinearity [47]. The following categorical covariates were dichotomized in order to minimize the number of variables in the multiple models: type of cancer (breast versus other), treatment intention (palliative versus curative, adjuvant, neo-adjuvant), treatment modalities (only chemotherapy versus chemotherapy with one or two additional treatment modalities), depression (no signs of depression versus mild, moderate, severe depression), performance status (level 0 versus ≥ I) and work type (sedentary versus physical, mixed).

In the final Cox regression models, a p-value < 0.05 was used as threshold for level of statistical significance. All analyses were performed using STATA 15.1 [48].

## Results

### Descriptive Data

Baseline characteristics of the study sample including 114 employees on full time sick leave at initiation of chemotherapy are shown in Table 1.

**Table 1:** Baseline sociodemographic, health-related, illness- and treatment-related, and work-related characteristics of a population of 114 sickness absent employees initiating chemotherapy for cancer

	N	
Age (years) (mean and SD, range)	114	51 (7.47), 25–62
Gender (n and %)	114	
Female		87 (76)
Man		27 (24)
Education level (n and %)	112	
None		11 (10)
Short		44 (39)
Medium		42 (38)
Long		15 (13)
Work type (n and %)	112	
Physical		31 (28)
Sedentary		47 (42)
Mixed		34 (30)
Perceived support from the work place (mean and SD)	107	8.50 (2.38)
Type of cancer (n and %)	114	
Female reproductive system		8 (7)
Breast		56 (49)
Lung incl. mesotheliomas		10 (9)
Urological incl. male reproductive system		5 (4)
Upper gastrointestinal		11 (10)
Colorectal		13 (12)
Cerebral and the central nervous system		5 (4)
Other		6 (5)
Treatment intention (n and %)	114	
Curative		8 (7)
Adjuvant		62 (54)
Neo-adjuvant		18 (16)
Palliative		26 (23)
Treatment modalities (n and %)	114	
Chemotherapy		90 (79)
Chemotherapy and one additional treatment modality		21 (18)
Chemotherapy and two additional treatment modalities		3 (3)
Time since diagnosis (days) (medium and iqr, range)	114	69.50 (49–94), 20–1132
Time since initiation of chemotherapy (days) (mean and SD, range)	114	33 (19.91), 0–84
Return to work self-efficacy (n and %)	114	
Low RTWSE		63 (55)
High RTWSE		51 (45)
Depression (n and %)	113	
No depression		80 (71)
Mild depression		24 (21)
Moderate depression		9 (8)
Severe depression		0 (0)
Fatigue (mean and SD)	113	19.03 (SD: 8.20)
Performance status (n and %)	113	
Level 0: Fully active, able to carry on all pre-disease performance without restriction		26 (23)
Level I: Restricted in strenuous activity but able to carry out work of a light nature		67 (59)
Level II: Capable of all self-care but unable to carry out any work activity		17 (15)
Level III: Capable of only limited self-care, in bed for more than 50% of the time		3 (3)
Level IV: Cannot carry out any self-care, totally confined to bed or chair		0 (0)

Frequency, percentage and range, mean and standard deviation or median and interquartile range

*RTWSE* return to work self-efficacy, *SD* standard deviation, *Iqr* interquartile range

During 15 months of follow-up, 63 (55%) participants had fully returned to work, 34 (30%) remained on sick leave (fulltime or part time), 7 (6%) had retired early, and 10 (9%) had died. Total time of follow-up was 5492 weeks. The median time to full RTW was 43.50 weeks (IQR: 27–65) for the complete sample.

### The Predictive Value of RTWSE on Full RTW

**Fig. 2:**
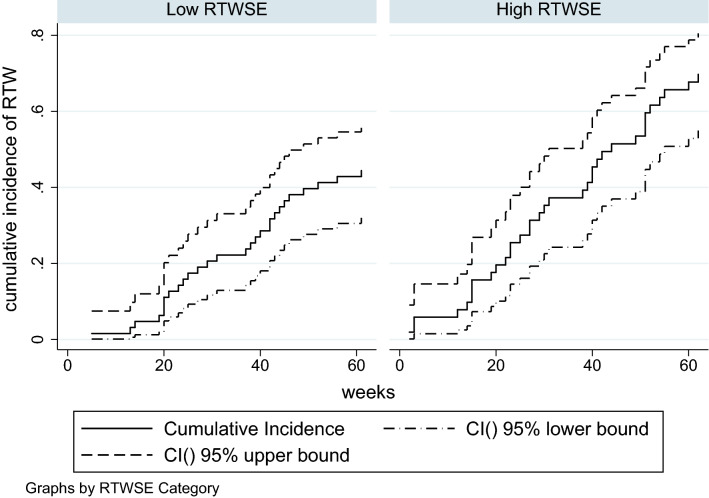
Aalen–Johansen cumulative incidence curves of full return to work for the low RTWSE group and the high RTWSE group. *RTWSE* return to work self-efficacy, *RTW* return to work, *CI* confidence interval

At baseline, 63 participants (55%) were categorized as having low RTWSE while 51 participants (45%) were categorized as high (Table 1). The median time to full RTW was shorter in the latter group, although the difference did not reach statistical significance (40 weeks (IQR: 23–60) compared to 45 weeks (IQR: 29–65), p = 0.058). Cumulative incidence curves of full RTW for the low and the high RTWSE groups, respectively, are shown in Fig. 2.

The unadjusted Cox proportional hazards regression showed a significant difference between the low and the high RTWSE-groups, with high RTWSE being associated with shorter time until full RTW (HR 1.84, 95% CI 1.12–3.03).

Based on the bivariate analyses, the following four variables were associated with weeks until full RTW at a level of p < 0.20; sex, treatment intention, depression, and perceived support from the workplace (Table 2). Hence, these variables were all included in the multiple Cox proportional hazards regression analyses as covariates.

**Table 2:** Bivariate associations between sociodemographic, health-related, illness- and treatment-related and work-related characteristics at baseline and weeks until full RTW during 15 months of follow-up in a population of 114 sickness absent employees initiating chemotherapy for cancer

Variable	N	HR	95% CI	P-value
RTWSE	114	1.84	1.12–3.03	0.016*
Gender				
Men	27	1	–	–
Women	87	0.58	0.32–1.02	0.059*
Age (years)	114	1.01	0.98–1.05	0.484
Educational level				
None	11	1	–	–
Short	44	1.20	0.50–2.92	0.680
Medium	42	0.76	0.31–1.89	0.556
Long	15	1.09	0.39–3.06	0.873
Work type				
Sedentary	47	1	–	–
Physical/mixed	65	1.33	0.80–2.23	0.274
Perceived support from work place (scale score)	107	1.14	0.99–1.30	0.061*
Type of cancer				
Breast	56	1	–	–
Other	58	1.28	0.78–2.12	0.326
Treatment intention				
Curative, adjuvant, neo-adjuvant	26	1	–	–
Palliative	88	0.31	0.13–0.78	0.013*
Treatment modalities				
Chemotherapy and no additional treatments	90	1	–	–
Chemotherapy + 1 or 2 additional treatments	24	1.22	0.68–2.22	0.506
Time since chemotherapy initiation (days)	114	1.00	0.99–1.02	0.567
Time since diagnosis (days)	114	1.00	0.99–1.00	0.273
Depression				
No depression	80	1	–	–
Symptoms of depression	33	0.58	0.32–1.07	0.082*
Fatigue (sum score)	113	1.00	0.97–1.03	0.985
Performance status				
Fully active without restrictions (Level 0)	26	1	–	–
Restricted in some way (level ≥ I)	87	0.85	0.48–1.51	0.584

Hazards ratios, confidence intervals and p-values of the bivariate Cox proportional hazards regression analyses

*HR* hazard ratio, *CI* confidence interval, *RTWSE* return to work self-efficacy

*Significant at p < 0.20

High RTWSE remained significantly associated with shorter time to full RTW when adjusting for sex, but after adding the illness- and treatment-related (model 3), and the health- and work place-related variables (model 4), high RTWSE did not remain significantly associated with shorter time to full RTW (Table 3). In the multivariate model (model 4), sex and treatment intention were the only variables significantly associated with RTW, indicating that being woman and receiving palliative treatment, respectively, were significantly associated with later RTW.

**Table 3:** Hazard ratios for returning to work during 15 months of follow-up associated with the baseline level of return to work self-efficacy in a population of 114 employees undergoing treatment for cancer, including the Hazard Ratios for the covariates (gender, treatment intension, depression, and perceived support form work place)

	Model 1 (unadjusted) (N = 114)			Model 2^a^ (N = 114)			Model 3^b^ (N = 114)			Model 4^c^ (N = 107)		
	HR	95% CI	p value	HR	95% CI	p value	HR	95% CI	p value	HR	95% CI	p value
Low return to work self-efficacy	1	**–**	**–**	1	**–**	**–**	1	**–**	**-**	1	**-**	**-**
High return to work self-efficacy	1.84	1.12–3.03	**0.016**	1.75	1.06–2.89	**0.030**	1.58	0.95–2.65	0.079	1.12	0.62–2.02	0.711
Gender												
Male				1	–	–	1	–	**–**	1	**–**	**–**
Female				0.63	0.36–1.13	0.124	0.36	0.19–0.68	**0.001**	0.30	0.15–0.60	**0.001**
Treatment intention												
Curative, adjuvant, neo-adjuvant							1	**–**	**–**	1	–	**–**
Palliative							0.20	0.07–0.52	**0.001**	0.15	0.05–0.44	**0.001**
Depression												
No sign of depression										1	**–**	**–**
Symptoms of depression										0.89	0.46–1.74	0.742
Perceived support from the work place												
Scale score*										1.13	0.98–1.30	0.085

Hazards ratios, confidence intervals and p-values of the unadjusted and the multivariate cox proportional hazards regression models

*HR* Hazard Ratio, *CI* Confidence interval

^a^Adjusted for gender

^b^Adjusted for gender and treatment intention

^c^Adjusted for gender, treatment intention, depression, and perceived support from the work place

*Scale score with higher scores referring to higher levels of perceived support

The VIF of the five independent variables (RTWSE, sex, treatment intention, depression and perceived support from the workplace) ranged from 1.14 to 1.27, indicating no multicollinearity.

## Discussion

### Main Findings

To our knowledge, this is one of only two studies examining the predictive value of RTWSE in a sample of employees undergoing treatment for cancer. Using a 15-month follow-up design and register-based data on RTW, a statistically significant positive association between RTWSE and returning full-time to work was found in the unadjusted regression model. However, the results did not remain statistically significant when taking into consideration a number of relevant covariates. In the multivariate model, only female sex and palliative treatment intention remained significant (negative) predictors of RTW. Of the total sample of 114 employees, 63 (55%) returned to full time work within the 15 months of follow-up, which is in accordance with previously observed RTW rates in populations of cancer patients, i.e., on average 62% (range 30–93%) 12–24 months after diagnosis [2, 5].

### Interpretation of Findings and Implications

RTWSE has previously been found to be predictive of RTW in populations of employees on sick leave due to both mental [19, 25] and musculoskeletal disorders [20, 23], in populations with all-cause sickness absence [24, 26] and within three [19, 20, 26], twelve [23, 25], and 24 months [24]. Surprisingly, the findings of the present study were not in line with these. This may be explained by the diagnostic differences of the study samples. Being on sick leave due to cancer may differ from being on sick leave due to other disorders, and the role of RTWSE as a determinant of RTW may be overshadowed by other factors when examined in employees with cancer. Thus, unlike the other sickness absent populations in which RTWSE has been observed to be predictive, other factors appear be more predictive of RTW in populations of cancer patients, i.e., female sex and palliative treatment as suggested by the results of the present study. The significant role of sex, treatment type and disease stage has previously been reported in systematic reviews as prognostic factors for RTW among cancer patients [1–3, 30, 32]. However, it must be noticed that the results regarding the significance of palliative treatment in the present study are based on a small sample, i.e., only 26 employees (23%) were undergoing palliative treatment of whom only five returned to work within the 15 months of follow-up.

In contrast to the present study, Wolvers et al. [29] found RTWSE to be predictive of RTW in employees with cancer. Keeping in mind that the participants in Wolver et al.’s study [29] were all in curative treatment, in physically good shape, and motivated for RTW, one might speculate that RTWSE is more likely to be predictive of RTW in populations of cancer patients in curative care who are motivated for RTW than in populations of cancer patients with a wider range of treatment intentions (i.e., curative, adjuvant, neo-adjuvant and palliative) who are therefore potentially less motivated for RTW. It is well-known that after being diagnosed with a life-threatening illness, life priorities may change [12–14], and it is possible that a subsample in the present study could have had an expectation of *being able to RTW* but *chose not to RTW* due to other life priorities. Approximately 50% of the sample in the present study were younger women with breast cancer who may have prioritized to stay home during treatment and for a longer time period due to family life and children living at home. Taking care of household tasks and/or children are reported as barriers to RTW in previous research [49]. Due to current Danish sickness absence legislation [36], receiving sickness absence compensation is possible for 52 weeks for many cancer patients, and prioritizing to stay home for 12 months was thus a possibility for these women. Likewise, the employees undergoing palliative treatment may also have had other life priorities beside RTW [13, 14, 49, 50]. Differences in sample characteristics with regard to treatment intention and motivation for RTW are therefore possible explanations as to why RTWSE failed to predict RTW in the present sample of employees with cancer but was predictive in the study by Wolvers et al. [29]. The majority of the population in Wolvers et al.’s study was women with breast cancer, too. Still, the possible difference between the two groups of women with breast cancer is the motivation for RTW, as all women in the study by Wolvers et al. were participating in an intervention aiming at increasing RTW. Motivation as a key factor in the RTW process of cancer patients has been reported in a meta-synthesis of qualitative studies of cancer patient's experiences of RTW [50]. According to social cognitive theory, SE and motivation are tightly connected. Motivation is defined as "a general construct that encompasses a system of self-regulatory mechanisms" [17] and consists of three main features: "Selection, activation, and sustained direction of behavior towards certain goals" [17]. These goals can also be called outcome expectancies and in cooperation with SE play a key role in human behavior [17]. Motivation directs behavior towards goals, but SE will still be the major basis for action; if the individual does not believe that he or she can attain the desired outcome, he/she will not engage in trying. So, if RTW is a desired outcome, and the individual believes that he or she can attain it, then the individual is likely to engage in trying. This is likely to be the case in the study of Wolvers et al. [29] in which all the participants participated in an intervention program aiming at RTW. On the contrary, if work is not a desired goal, the individual will not engage in a behavior that leads towards that goal, irrespective of their RTWSE. This may explain the divergence between the results of the present study and the study of Wolvers et al. [29]. Examining motivation for RTW as an effect modifier in the association between RTWSE and RTW in populations of employees with cancer is recommended in future research. It is possible that in samples of cancer patients who are motivated for RTW, RTWSE might be predictive, but in samples of patients less motivated, the predictive value is reduced.

### Implications for Practice

Being predictive of RTW, a RTWSE questionnaire could help identifying sickness absentees in risk of prolonged sickness absence [24] and thereby be a relevant tool in occupational rehabilitation practice. However, the predictive value of RTWSE could be different when struggling with cancer compared with other diseases. This should be taken into consideration by stakeholders in occupational cancer rehabilitation prior to applying these tools. Based on the present findings and the findings of Wolvers et al. [29], it is hypothesized that RTWSE may be predictive in some groups of employees with cancer and not in others and that motivation for RTW may be an important aspect regarding the predictive value. This hypothesis should be investigated further. Identifying the characteristics of the cancer patients, for whom the RTWSE is predictive is useful within an occupational rehabilitation context. Patients with cancer often feel on their own when handling questions regarding work life and express a wish for guidance from the hospital or their employers [49, 51, 52]. However, many health professionals hesitate to talk about work-related issues with these patients because they find it difficult and some even unethical to do so [53, 54]. The RTWSE questionnaire may help to identify the patients in most need of help and further be a tool for healthcare professionals and other stakeholders to identify the most profound and difficult aspects of RTW for the individual patient and to structure the conversation regarding work-related issues. However, the first step is to identify for whom the RTWSE questionnaire is predictive. The findings of the present study add to the existing evidence by suggesting that RTWSE might only be predictive for some groups of cancer patients.

### Methodological Considerations

In the present study, the RTWSE-19 was used as it is the only RTWSE questionnaire culturally adopted, translated and validated in Denmark [26]. Although it is possible that RTWSE-19 is less predictive of RTWSE than the other available instruments (RTWSE-11 [19, 25, 29] and RTWSE-10 [23], the RTWSE-19 questionnaire has been found to be predictive of actual RTW in populations of sickness absentees with low back pain [20] and various diseases [26]. However, it is well known, that the psychometric properties of a measurement tool may change if the scale is used in another population [55]. The authors of the present study have also conducted at validation study of the return to work self-efficacy questionnaire in a population of employees undergoing treatment for cancer (paper under review). This study confirms the validity of the RTWSE-19 among cancer patients. RTWSE-19 is thus, to the best of our knowledge, the only RTWSE questionnaire, which has been validated in a cancer population. Yet, the divergent finding of the present study might be explained by using the scale in a population for which it was not developed. As the RTWSE-11 was developed for employees on sick leave due to mental health problems [19], which are also prevalent in cancer patients [5], it is possible, that the RTWSE-11, used by Wolvers et al. [29] to confirm the predictive value of RTWSE in a cancer population, may be more predictive of RTW in cancer populations than the RTWSE-19.

RTWSE-19 includes three subscales. In the present study, the predictive value was examined according to the total scale and not the three subscales. The RTWSE-19 questionnaire was developed from qualitative interviews and the concept RTWSE was found to include all three aspects [20, 21], that is, all three aspects are of significance for the RTWSE of an individual. Therefore, regarding the predictive value, we chose to analyze the total scale score only. This is in line with the original article of Shaw et al. [20].

RTWSE as independent variable has been defined differently in previous studies. In the present study, the analyses of the predictive value of the RTWSE were done based on a dichotomization of the participants into a low vs. a high RTWSE group. A cut-off point of 7.5 was chosen, based on the original cut-off value found by Shaw et al. [20] for the upper tertile group (i.e., high RTWSE). In the present study, we chose dichotomization due to: (1) dichotomization at the upper tertile was in line with the design in the previous Danish validation study examining the predictive value of RTWSE-19 [26], (2) a reduction of variables in the regression models was desirable in order to minimize the risk of overfitting the multiple regression models [56]. Keeping in mind our small sample size, a dichotomization was thus more sound than splitting in three, and (3) in the clinical setting, one cut-off value was assumed simpler to apply. Additional analyses of the present data showed that the results did not change when analyzing the predictive value of RTWSE according to low, moderate and high RTWSE compared to the applied dichotomization.

Dichotomizations of the RTWSE scales at the upper tertile (RTWSE-19) [26], the upper quartile (RTWSE-11) [24], or at the median (RTWSE-11) [25] have been used in previous studies measuring and confirming the predictive value of RTWSE. In other studies, the total RTWSE scale and subscale scores have been used as independent variables (RTWSE-11 and RTWSE-10) [19, 23, 29]. Brouwer et al. [23] further defined changes in the RTWSE-10 scores over time as the independent variable with the conclusion that improvements in RTWSE from baseline to 6 months were predictive of work status at 12 months. However, despite these differences in the operationalization of RTWSE, all of the previous studies have found RTWSE to be predictive [19, 23–26, 29]. Thus, defining RTWSE in a specific way does not seem to be the essential factor for finding RTWSE as a concept to be predictive and the choice of dichotomizing at the upper tertile in the present study does not seem a likely explanation for the non-significant findings in the present study. Future research could preferably include changes in RTWSE scores as predictor variable too when examining the predictive value of RTWSE in populations of employees with cancer. Including change in RTWSE as the independent variable might contribute with knowledge regarding the possible importance of increasing RTWSE during cancer treatment.

### Strengths and Limitations

The present study has several strengths. First, the dependent variable ‘time to full RTW’ was obtained by register data, ensuring 100% follow-up. Second, all independent variables were measured with validated instruments. Thirdly, including employees undergoing treatment with various intentions adds new knowledge within this research area, as this has not been examined before.

A number of limitations should also be mentioned. First, a relatively large number of patients were non-responders to the initial invitation to participate, i.e., the large number of "contact information sheets not returned (unknown reasons)" (*n* = 416). As information regarding these non-responders was impossible to obtain, comparisons between the non-responders and the responders regarding sociodemographic and illness- and treatment-related variables was not possible. However, it could have informed us of selection bias. Patients participating in research programs tend to have better functioning and higher socio-economic status compared to non-participants [57–59], hence, the risk of selection bias is present. Another limitation is the small sample size as it increases the risk of overfitting the multiple regression models [56]. The results of the present study should thus be interpreted with caution and larger samples are recommended for future studies. Finally, anxiety was not included as a covariate in the present study. It could, however, have been a relevant factor to include, as anxiety is included as covariate in the study of Volker et al. [24], and has proven associated with RTW in cancer patients [60]. Thus, anxiety could be a relevant as a covariate in future research examining the predictive value of RTWSE in cancer patients.

## Conclusion

The present study did not confirm the hypothesis of a predictive value of RTWSE regarding returning full-time to work within 15 months, and hence, did not support previous studies from non-cancer as well as cancer settings. The findings in the present study suggested that RTWSE may be a less important predictor of RTW compared with sex and treatment intention. Furthermore, it is possible that motivation for RTW is a possible modifier of the association between RTWSE and RTW, a hypothesis which remains to be investigated in future studies.

## Data Availability

The datasets analyzed during the current study are available from the corresponding author on reasonable request.
